# Infection of tendon sheaths, joints, bursae, soft tissue, and tendon rapture by brucella: A case report

**DOI:** 10.1002/ccr3.8157

**Published:** 2023-11-20

**Authors:** Mehrangiz Zangeneh, Kiana Rezvanfar, Yasamin Khosravani‐Nejad, Yazdanali Faghani, Manije Dezfulinejad, Masoud Saadat Fakhr, Parastoo Ghorbani

**Affiliations:** ^1^ Department of Infectious Diseases, Amir‐al‐Momenin Research Center, Tehran Medical Sciences Branch Islamic Azad University Tehran Iran; ^2^ Department of Infectious Diseases, Buali Research Center, Tehran Medical Sciences Branch Islamic Azad University Tehran Iran; ^3^ Department of Infectious Diseases, Farhikhtegan Research Center, Tehran Medical Sciences Branch Islamic Azad University Tehran Iran; ^4^ Department of Thoracic Surgery, Farhikhtegan Research Center, Tehran Medical Sciences school Branch Islamic Azad University Tehran Iran; ^5^ Department of Internal Medicine, Faculty of Medicine Mazandaran University of Medical Science Sari Iran

**Keywords:** arthritis, brucellosis, tendon tear

## Abstract

**Key Clinical Message:**

Arthritis is one of the main presentations of chronic brucellosis, but bursitis and tendon rupture are also caused by brucellosis. Therefore, brucellosis should be considered in the differential diagnosis of arthritis, bursitis, and tendon rupture by physicians. In addition, early diagnosis and treatment are very important in the prevention of disability.

**Abstract:**

Brucellosis is a zoonotic disease common in the Middle East. Manifestations of acute disease are fever, sweating, myalgia, and arthralgia. However, bone joint involvement occurs in 10%–85% of patients, and sacroiliac involvement occurs in up to 80% and vertebral joint involvement in up to 54%. A 57‐year‐old woman was admitted to the hospital of Islamic Azad University on February 26, 2021, with a history of one‐month pain and limited movement of the right shoulder joint with fever for surgery of the shoulder ligament. The standard agglutination titer (Wright) for brucellosis at first was 1/640 and then increased. MRI of the shoulder showed a supraspinatus tendon and anterosuperior labral of the glenoid labrum tear. Although the patient was a candidate for shoulder joint ligament surgery, with a diagnosis of brucellosis, the treatment of brucellosis was prescribed, symptoms disappeared with anti‐brucellosis antibiotic therapy without surgery, and the patient recovered. Supraspinatus tendon and antero superior labral of glenoid labrum tear of the shoulder joint in brucellosis is generally very rare. Failure or delay in the treatment of brucellosis can cause ligament rupture or joint disability. Sometimes, there are no symptoms except osteoarticular manifestations in brucellosis; therefore, brucellosis should be one of the differential diagnoses in osteoarticular diseases.

## Graphical abstract

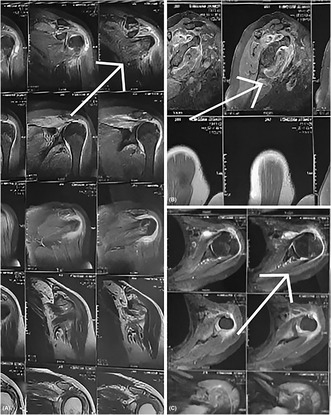

## 1 INTRODUCTION

Brucellosis is a worldwide zoonotic disease, especially in Asia and Africa.^1^ Four of the 12 Brucella species are pathogenic to humans, although the severity of infection is lower.^2^ Brucellosis is one of the causes of long fevers with a variety of clinical symptoms in endemic areas; however, brucellosis patients do not always have a fever. In some countries, brucellosis prevalence exceeds 10 cases per 100,000 people.^3^ In Iran, the western and northwestern regions are endemic areas for brucellosis, and villages are more affected than cities due to working conditions.^4^ Brucella is transmitted to humans in different ways, such as by eating undercooked meat and unpasteurized dairy products contaminated with Brucella. It is also transmitted by inhalation of infected particles, or entry of bacteria through skin lesions and mucous membranes in people in close contact with infected animals.^1^ Brucellosis treatment takes time, so treating brucellosis is difficult. It is important to note that antibiotic therapy for brucellosis takes several weeks to treat and prevent its progression. Antibiotics such as doxycycline, rifampin, tetracycline, and cotrimoxazole are common drugs used to cure brucellosis. Brucella infections can affect any organ. The spleen, liver, testis, bone marrow, and joints are commonly infected organs. Brucellosis clinical manifestations include fever, myalgia, arthralgia (especially in children), and neurological symptoms (mostly in adults). Nevertheless, chills, rigor, and night sweats are also nonspecific symptoms.^4^, ^6^ Bone joint involvement also occurs in 10%–85% of patients. Sacroiliac joints up to 80% and vertebral joints up to 54% are the most common sites of involvement. However, ligament and tendon tears in brucellosis are rare. However, the definitive clinical signs of this disease have not yet been accurately identified, and new brucellosis symptoms are always reported. Therefore, careful study of the disease symptoms is always necessary for early diagnosis and timely treatment. This study reported a 53‐year‐old woman with brucellosis with glenohumeral joint effusion, rotator interval ligament edema and thickening, supraspinatus tendon tears, and anterosuperior labral tears of the glenoid labrum. She recovered with antibiotic therapy for 4 months without surgery.

## 2 CASE PRESENTATION

A 57‐year‐old woman without preexisting diseases was admitted to the Islamic Azad University Hospital in Tehran on February 29, 2021, with complaints of pain and limited movement in the right shoulder joint. In addition, she had a history of arthralgia in other joints, especially the knees, and low back pain. This started approximately 3.5 months before presentation. Right shoulder pain and limited mobility developed 2 months later. The progression from the lower to upper body over 3 months is characteristic of brucellosis spread.

Additionally, she had loss of appetite, 4 kg weight loss (8.8 lb), sometimes low‐grade fever and weakness 3 months prior. At the beginning of the disease, the patient had low back pain, knee and other joint arthralgia, and then, the pain transferred to the right shoulder joint. The patient's medical past history was hyperlipidemia and lumbar disk surgery 20 years ago.

The patient, when admitted to the hospital orthopedic department for shoulder ligament rapture surgery, was not ill and had no other signs or symptoms. After infectious disease consultation, for the patient, abdominal ultrasound, chest CT scan, whole blood test, wright, coombs wright, 2ME, and echocardiography (TTE) were ordered, and all results were normal except wright, coombs wright, and 2ME (Tables 1 and 2). Brucella IgM positivity results showed that the patient acquired brucellosis recently. The differential diagnosis was septic arthritis and rheumatoid arthritis. Key features of septic arthritis that differ from this case are the acute onset of severe joint pain, fever, and positive joint fluid cultures. Rheumatoid arthritis typically involves smaller joints, is bilateral, exhibits morning stiffness, and is associated with elevated inflammatory markers. Blood cultures were negative, making septic arthritis unlikely. The patient was rheumatoid factor negative and anti‐CCP negative, arguing against rheumatoid arthritis. The dramatic response to antibiotics also supports brucellosis as the correct diagnosis.

**TABLE 1:** Serological and immunological tests of brucellosis on February 29th, 2021.

Laboratory test	Result	Unit	Reference interval
WBC	8000	10^3^/mm	4.1–11
HGB	11.2	g/dL	12.5–15
C.R. P	30	mg/L	<10 mg/L
ESR 1 h	92	mm	0_30
Wright	1/1280	Titer	Negative <1/80 Positive >1/80
Coombs wright	1/640	Titer	Negative Up to 1/40
2ME	1/160		Negative
Anti‐CCP	Negative	Unit	≤1/20 EU/mL
HLA b27	Negative	Mg/L	<10 mg/L
RF	Negative	Unit	0–20 IU/mL
ANA	1/80	Au/mL	≤1/160 positive
Vitamin D	46	ng/mL	Normal

**TABLE 2:** Serological tests of brucellosis 4 months after treatment.

Serology test	Result	Unit	Reference interval
C.R.P	Negative	mg/L	Negative
ESR 1 h	12	mm	0–30
Wright	1/160	Titer	Negative <1/80 Positive >1/80
Coombs wright	1/80	Titer	Negative Up to 1/40
2ME	Negative	Titer	Negative
Brucella IgM	2.9	ELISA	>11 positive <9 negative
Brucella IgG	17.1	ELISA	>11 positive <9 negative

**FIGURE 1:**
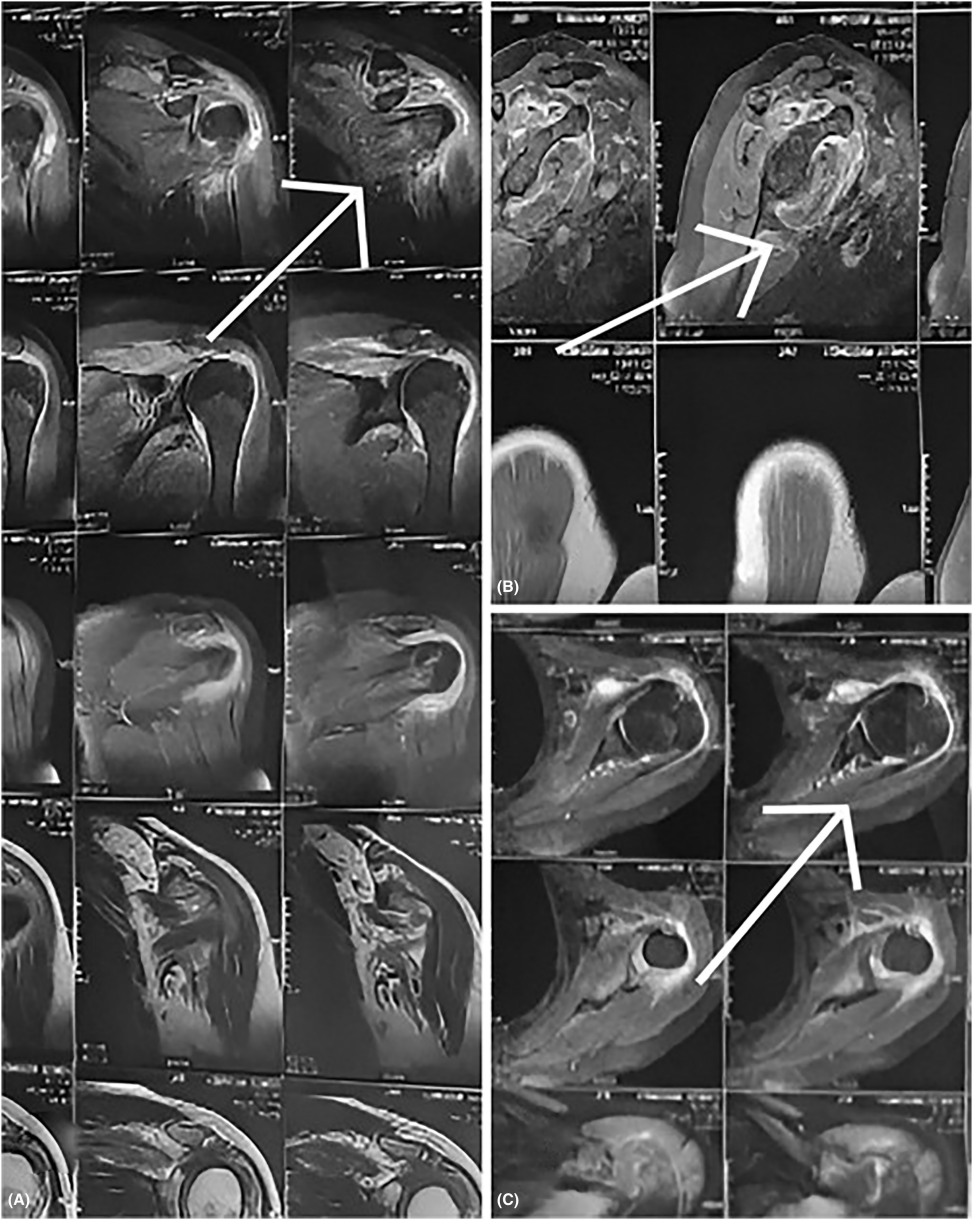
MRI related to table number two, which shows a high‐grade tear with muscle edema and strain in the supraspinatus tendon (A), an anterosuperior labral tear in the glenoid labrum (B), and a partial tear of the IGHL glenohumeral ligaments (C).

According to the MRI results of the right shoulder joint (Table 3, Figure 1), the glenohumeral joint had mild to moderate effusion, soft tissue in the rotator interval had edema and thickening, the supraspinatus tendon and anterosuperior labral of the glenoid labrum had torn, the subacromial‐subdeltoid bursal had moderate loculated effusion, and the acromial clavicular joint had mild DJD with impingement. The patient was a candidate for surgery on the right shoulder ligaments, but infectious disease specialists decided to treat brucellosis instead of surgery. After the diagnosis of brucellosis, treatment with cotrimoxazole (trimethoprim‐sulfamethoxazole) (400/80 mg bid), doxycycline (100 mg bid), pantoprazole (40 mg daily), and diclofenac sodium (100 mg bid) was prescribed.

**TABLE 3:** MRI in the sample with brucellosis on February 26th, 2021.

Glenohumeral joint effusion	Mild to moderate
Supraspinatus tendon	High‐grade tear with muscle edema and strain
Subcapularis tendon	Intact
Infraspinatus tendon	Intact
Biceps brachii tendon	Intact
Teres minor tendon	Intact
Glenohumeral ligaments	Partial tear of IGHL
Coracohumeral and caraco‐acromial ligament	Normal
Rotator interval	Soft tissue edema and thickening
Glenoid labrum	Anterosuperior labral tear
Bones and marrow	Normal
Neurovascular bundles	Intact
Joint capsule	Intact
Subacromial‐subdeltoid bursal effusion	Moderate loculated effusion
Articular cartilage	Intact
Acromial clavicular joint	Mild DJD with type III acromion and mild subacromial impingement
Muscles and soft tissues	Intact

During 6 weeks of antibiotic treatment, the patient complied fully with the regimen. She avoided lifting heavy objects or excessive shoulder exertion to rest the joint. Her activities were limited to basic self‐care and light household duties. Physiotherapy was not pursued during this 3‐month symptomatic period prior to presentation. Home exercises were recommended but not consistently followed by the patient due to pain.

The patient visited after 3 months of treatment on June 1, the pain and shoulder movement improved, and one more month of medication continued to 4 months. Table 2 shows test results after brucellosis treatment. After 4 months of medication, there was no shoulder pain or movement restriction in the right shoulder, and the patient did not need surgery.

## 3 DISCUSSION

Brucellosis is a worldwide zoonotic disease. Brucella transmits to humans primarily through the consumption of unpasteurized dairy products, inhalation of infected particles, and close contact with infected animals.^5^ Brucella causes disease in any organ, and the clinical symptoms of brucellosis include fever, night sweats, joint and muscle pain, and complications in any organ, such as the neurologic, skeletal, cardiovascular, and genitourinary systems.^4^, ^6^ Arthritis is a common complication of brucellosis, but tendon tears are a very rare complication of brucellosis. In our study, the pain first began from the lower back and knee and then migrated to the waist and then to the right shoulder, eventually causing tendon rupture and movement limitation of the shoulder. Serologic tests showed chronic brucellosis infection, and after antibiotic treatment for 4 months, the patient's clinical symptoms recovered, and serologic tests improved. Iran is one of the endemic regions with a high prevalence of brucellosis among humans and animals, which is mostly due to Brucella melitensis.^7^ The patient resides in a rural village with exposure to potentially contaminated dairy products from sheep and goats. Raw milk consumption is a key exposure in this setting. Brucella meltiness is the main causative species in the region. In a study by Dr. Ebrahimpour et al. in 2017, of the 464 patients studied, 75.4% had arthritis and 52% had peripheral arthritis, among whom 31.9% had knee arthritis and 11.9% had hip arthritis. This study showed that peripheral arthritis is the most common type of arthritis in patients with brucellosis.^8^ Brucella arthritis without treatment can cause joint infection or bone damage and disability.^9^ Therefore, according to our results, delays in the diagnosis and early treatment of brucellosis cause joint disability. In our case report, the time between the onset of low back pain, knee arthralgia, and shoulder disability was 3 months. One of the main reasons for misdiagnosis or late diagnosis of brucellosis is the similarity of brucellosis manifestations to those of other infectious diseases, such as influenza, Yang fever, or malaria, which leads to serious joint damage.^10^, ^11^ In a similar case reported by Fe‐sheng Wang et al. on February 6, 2021, a 26‐year‐old man presented with right shoulder pain and movement limitation without fever or night sweats and other joint arthralgia. Acromial bursitis was diagnosed due to brucellosis, and after 6 weeks of antibiotic therapy, the patient recovered without symptoms.^12^ In a study by Almajid on February 26, 2017, a 43‐year‐old man presented with swelling and pain in his right elbow, right leg, and low back without fever. The patient had olecranon bursitis, and after 3 months of antibiotic therapy, she did not show any symptoms.^13^ Arvind Mishka et al. reported in 2018 a rare case in which a 16‐year‐old boy presented with migratory joint pain and limited movement from the knee to the right shoulder and then the right wrist. After receiving corticosteroids, the pain increased, the patient developed a fever, and serologic diagnostic tests for brucellosis were positive. The patient, after 6 weeks of antibiotic therapy, recovered.^14^

## 4 CONCLUSIONS

According to this study and other similar studies, arthritis and bursitis are the main presentations of chronic brucellosis, but tendon rupture has also been reported. Therefore, brucellosis should be considered in the differential diagnosis of arthritis, bursitis, and tendon rupture by physicians. In addition, early diagnosis and treatment are very significant in disability prevention.

## AUTHOR CONTRIBUTIONS

**Mehrangiz Zangeneh:** Conceptualization; investigation. **Kiana Rezvanfar:** Writing – original draft; writing – review and editing. **Yasamin Khosravani‐Nejad:** Software; visualization. **Yazdanali Faghani:** Methodology; visualization. **Manije Dezfulinejad:** Formal analysis; investigation. **Masoud Saadat Fakhr:** Software. **Parastoo Ghorbani:** Project administration; writing – review and editing.

## FUNDING INFORMATION

None.

## CONFLICT OF INTEREST STATEMENT

None.

## CONSENT

Written informed consent was obtained from the patient to publish this report in accordance with the journal's patient consent policy.

## Data Availability

The data are available with the correspondence author and can be reached on request.
